# Using Brain Oscillations and Corticospinal Excitability to Understand and Predict Post-Stroke Motor Function

**DOI:** 10.3389/fneur.2017.00187

**Published:** 2017-05-10

**Authors:** Aurore Thibaut, Marcel Simis, Linamara Rizzo Battistella, Chiara Fanciullacci, Federica Bertolucci, Rodrigo Huerta-Gutierrez, Carmelo Chisari, Felipe Fregni

**Affiliations:** ^1^Neuromodulation Center, Spaulding Rehabilitation Hospital, Harvard Medical School, Boston, MA, USA; ^2^Institute of Physical and Rehabilitation Medicine, University of Sao Paulo Medical School, Sao Paulo, Brazil; ^3^Unit of Neurorehabilitation, University Hospital of Pisa, Pisa, Italy; ^4^Faculty of Medicine, National Autonomous University of Mexico, Mexico City, Mexico

**Keywords:** stroke, motor function, recovery, EEG, beta oscillations, biomarker, Fugl-Meyer, transcranial magnetic stimulation

## Abstract

What determines motor recovery in stroke is still unknown and finding markers that could predict and improve stroke recovery is a challenge. In this study, we aimed at understanding the neural mechanisms of motor function recovery after stroke using neurophysiological markers by means of cortical excitability (transcranial magnetic stimulation—TMS) and brain oscillations (electroencephalography—EEG). In this cross-sectional study, 55 subjects with chronic stroke (62 ± 14 yo, 17 women, 32 ± 42 months post-stroke) were recruited in two sites. We analyzed TMS measures (i.e., motor threshold—MT—of the affected and unaffected sides) and EEG variables (i.e., power spectrum in different frequency bands and different brain regions of the affected and unaffected hemispheres) and their correlation with motor impairment as measured by Fugl-Meyer. Multiple univariate and multivariate linear regression analyses were performed to identify the predictors of good motor function. A significant interaction effect of MT in the affected hemisphere and power in beta bandwidth over the central region for both affected and unaffected hemispheres was found. We identified that motor function positively correlates with beta rhythm over the central region of the unaffected hemisphere, while it negatively correlates with beta rhythm in the affected hemisphere. Our results suggest that cortical activity in the affected and unaffected hemisphere measured by EEG provides new insights on the association between high-frequency rhythms and motor impairment, highlighting the role of an excess of beta in the affected central cortical region in poor motor function in stroke recovery.

## Introduction

Stroke is a leading cause of morbidity, mortality, and disability worldwide (1, 2). Among the sequels of stroke, motor impairment is one of the most relevant, since it conditions the quality of life of patients, it reduces their capability to perform their daily activities and it impairs their autonomy (3). Despite the advancements of the acute stroke therapy, patients require an intensive rehabilitation program that will partially determine the extent of their recovery (4). These rehabilitation programs aim at stimulating cortical plasticity to improve motor performance and functional recovery (5). However, what determines motor improvement is still unknown. Indeed, finding markers that could predict and enhance stroke recovery is still a challenge (6). Different types of biomarkers exist: diagnostic, prognostic, surrogate outcome, and predictive biomarkers (7). The identification of these biomarkers is critical in the management of stroke patients. In the field of stroke research, great attention has been put to biomarkers found in the serum, especially in acute care. However, research on biomarkers of stroke recovery is still limited, especially using neurophysiological tools.

A critical research area in stroke is to understand the neural mechanisms underlying motor recovery. In this context, neurophysiological techniques such as transcranial magnetic stimulation (TMS) and electroencephalography (EEG) are useful tools that could be used to identify potential biomarkers of stroke recovery. However, there is still limited data to draw further conclusions on neural reorganization in human trials using these techniques. A few studies have shown that, in acute and sub-acute stage, stroke patients present increased power in low frequency bands (i.e., delta and theta bandwidths) in both affected and unaffected sides, as well as increased delta/alpha ratio in the affected brain area; these patterns being also correlated to functional outcome (8–11). Recently, we have identified that, besides TMS-indexed motor threshold (MT), an increased excitability in the unaffected hemisphere, coupled with a decreased excitability in the affected hemisphere, was associated with poor motor function (12), as measured by Fugl-Meyer (FM) [assessing symptoms severity and motor recovery in post-stroke patients with hemiplegia—Fugl-Meyer et al. (13); Gladstone et al. (14)]. However, MT measurement is associated with a poor resolution as it indexes global corticospinal excitability. Therefore, combining this information with direct cortical measures such as cortical oscillations, as measured by EEG, can help us to understand further neural mechanisms of stroke recovery.

To date, there are very few studies looking into EEG and motor recovery. For that reason, we aimed, in the present study, to investigate the relationship between motor impairment, EEG, and TMS variables. To do so, we conducted a prospective multicenter study of patients who had suffered from a stroke, in which we measured functional outcome using FM and performed TMS and EEG recordings. Based on our preliminary work, we expected to identify changes in interhemispheric imbalances on EEG power, especially in frequency bands associated with learning, such as alpha and beta bandwidths.

## Materials and Methods

### Database

Our database was built from two separate studies conducting EEG analysis and motor impairment assessments in stroke subjects in order to understand neurophysiological signatures of motor recovery. We conducted an individual patient meta-analysis as to understand the combined data from these two studies. This database included baseline data of 55 subjects with a clinical diagnosis of stroke. Thirty-five subjects (62 ± 13 years old; 26 ± 11 months post-injury; 16 left stroke; 29 ischemic stroke, 15 women) were enrolled in Brazil and 20 (62 ± 15 years old; 42 ± 68 months post-injury; 11 left stroke; 20 ischemic stroke, 2 women) in Italy. These studies were approved by the ethical committee of both institutions and patients signed the respective informed consent to participate in this study.

The inclusion criteria were: (1) age over 18 years, (2) clinical and neuroimaging-based diagnosis of stroke, (3) 6 months since the stroke, (4) clinically stable, and (5) stroke subjects with lower and upper limb impairment with minimal movement of the paretic upper limb, such as at least 10° of active wrist extension. The exclusion criteria were: (1) Mini-Mental Examination score lower than 24, (2) more than one stroke event, (3) psycho-affective disorders that prevented adherence to treatment, and (4) joint damage and pain or deformities that affected member that makes impossible the implementation of the therapy (5) contra-indication to TMS (15).

Note that the sample from Brazil has been already analyzed in a previous study (12).

Demographic data for both centers are presented in Table 1.

**Table 1:** **Demographic characteristics of the two datasets**.

	Center 1 (*n* = 35)	Center 2 (*n* = 20)
Age	62 ± 13.5 years	62.5 ± 15.5 years
Gender (%)	15 (43) women	2 (10) women
20 (57) men	18 (90) men
Affected hemisphere (%)	16 (46) left	11 (55.5) left
19 (54) right	9 (44.5) right
Type of stroke (%)	29 (83) ischemic	20 (100) ischemic
6 (17) hemorrhagic	0 (0) hemorrhagic
Time since injury	26 ± 11 months	43 ± 68 months
Fugl-Meyer	51.6 ± 7.9	35.6 ± 22.9
WMFT time	179.2 ± 211.9	675.5 ± 749.8
WMFT funct.	3.54 ± 0.55	39.6 ± 29.5
MT AH	58.7 ± 17.2	72.2 ± 16.1
MT UAH	51.8 ± 13.4	54.8 ± 10.5

*AH, affected hemisphere; MT, motor threshold; UAH, unaffected hemisphere; WMFT, Wolf Motor Function Test*.

### Assessments

The main goal of this study was to correlate a clinical assessment, namely the FM score, with two neurophysiological markers: MT and EEG.

#### Fugl-Meyer

Fugl-Meyer is a behavioral scale assessing motor impairment, balance, sensation, and joint functioning in post-stroke patients (13, 14).

#### Transcranial Magnetic Stimulation (TMS)

Single-pulsed TMS data were acquired by using 70 mm figure-8 coils in both centers (BiStim^2^, Magstim Company Ltd. in Brazil and MagProX100 MagOption stimulator, MagVenture in Italy). The same methodology for obtaining resting MT was employed in both sites. MT was defined as the lowest intensity of the stimulus that elicited a motor-evoked potential (MEP) with an amplitude of at least 50 µV in at least 50% of trials. The first dorsal interosseous muscle was used to obtain MEPs. MT was recorded electroencephalography in every patient, for both hemispheres. Patients who did not have measurable MT were excluded from analysis.

#### Electroencephalography (EEG)

EEG data were acquired by using a 128-channel EEG cap (Acti-Champs, PyCorder, Brainvision LLC^®^) in Brazil and a 62-channel EEG cap (Micromed system) in Italy. EEGs were recorded eyes closed for a minimum of 6 min in both centers. Data were sampled at a rate of 250 Hz, amplified, and filtered using a bandpass of 0.1–70 Hz. For offline analysis, we used a low-pass cut filter of 40 Hz and high pass of 1 Hz, followed by manual artifact detection and rejection by a blinded assessor. As resting state EEG recorded eyes closed were relatively clean, we did not perform ICA and removed noisy epochs manually. Power was calculated using EEGLab (16) and MATLAB (MATLAB R2012a; The MathWorks Inc., Natick, MA, USA). Fast Fourier transformation (averaged windows of 5 s with 50% overlap) was used to calculate power (μV^2^) for the following EEG bands: theta (4–8 Hz) and alpha (8–13 Hz) and the sub-bands: low alpha (8–10 Hz), high alpha (10–13 Hz), low beta (13–20 Hz), and high beta (21–30 Hz). Adjacent electrodes were selected and averaged to represent frontal (F1, F2, F3, F4, F5, F6, AF3, AF4, FC3, FC4), central (C1, C2, C3, C4, C5, C6), and parietal (P1, P2, P3, P4, P5, P6) areas in affected and unaffected hemispheres.

### Statistical Analysis

All analyses were performed with Stata Statistical Software 13 (StataCorp. 2013).

We conducted regression models to understand which neurophysiological variables could explain the variability of FM scores. The following baseline characteristics were assessed to be included in multivariable analyses: (1) MT for the affected and unaffected upper limb, (2) EEG power for theta, alpha, and beta bandwidths in the frontal, central, parietal, and occipital areas. We then included the following clinical variables: (1) age, (2) months since injury, (3) side of lesion and, (4) type of stroke, (5) gender. Finally, we forced center (Brazil and Italy) into the model as to assess potential sources of variability.

We initially performed univariate linear regression analyses in which the outcome variable was FM scores and the independent variable was EEG variable or MT. Affected and unaffected hemispheres were tested separately, as well as combined for EEG.

To determine the best EEG variables to include in the model, we used a step-wise linear regression with a backward elimination approach, using a significance level set at α = 0.05. We included EEG power variable of all bandwidths for central, both affected and unaffected hemispheres.

We determined the effects of confounders in these models by adding independent variables and assessing whether the β coefficient changed by more than 10%.

We then performed multivariate regression analyses using MT and EEG variables in the same model. Different models were tested: model 1 included FM and high-beta central in the unaffected hemisphere; model 2 included FM, high-beta central in the unaffected hemisphere, and high-beta central in the affected hemisphere; and model 3 included FM, high-beta central in the unaffected hemisphere and high-beta central in the affected hemisphere and MT of the affected upper limb.

Because this was an exploratory study and to minimize the risk of type II errors, no correction for multiple comparisons was done.

## Results

Fifty-five patients were enrolled in this study (62 ± 14 years old, 32 ± 42 months since injury, 27 left stroke, 17 women). Demographic and clinical characteristics are reported in Table 1.

### Univariate Analysis

We first conducted univariate analysis to identify variables that were associated with motor impairment as measured by FM. We assessed the effect of MT (affected and unaffected hemispheres) and EEG power spectrum variables (as defined in Section “Statistical Analysis”).

#### FM and MT

There was a significant main effect of MT in the affected hemisphere (*p* < 0.0001, β coeff. = −0.491, adj *R*^2^ = 0.315), indicating that higher MT is linked to worse motor function. The MEPs could not be elicited in the affected hemisphere in three patients; therefore, they were excluded from the analyses. There were no effects for the unaffected hemisphere (*p* = 0.126, β coeff. = −0.284, adj *R*^2^ = 0.026).

#### FM—EEG Power Spectrum

Power spectrum in high-frequency bands, mainly high alpha, low beta, and high beta in affected and unaffected hemispheres was associated with FM, indicating that high-power spectrum is associated with better motor function. The results are summarized in Table 2.

**Table 2:** **Results for univariate linear regression analyses in which the outcome variable was Fugl-Meyer and independent variable was EEG power**.

Power bandwidth	Affected hemisphere			Unaffected hemisphere		
	Frontal	Central	Parietal	Frontal	Central	Parietal
Theta	0.57	0.769	0.864	0.774	0.375	0.497
Alpha	0.069	0.068	**0.036**	0.094	**0.042**	**0.043**
Low alpha	0.119	0.112	0.142	0.165	0.061	0.152
High alpha	**0.046**	**0.041**	**0.007**	0.066	**0.038**	**0.009**
Low beta	**0.045**	0.090	**0.029**	0.053	**0.026**	**0.024**
High beta	**0.022**	0.113	**0.030**	**0.021**	**0.012**	**0.016**

*Bold numbers stand for significant results*.

### Predictors

When factors (all bandwidths for affected and unaffected hemispheres—central regions) were entered in a step-wise logistic regression analysis, only the high-beta central for both unaffected (*p* = 0.005; β coeff. = 172.43) and affected hemispheres (*p* = 0.046; β coeff. = −101.52) maintained a significant association with FM (adj. *R*^2^ = 0.148).

### Multivariate Analysis

We included the two main variables identified using the method exposed above in the model with FM as the main predictor.

In the univariate model (model 1), there was a significant effect of high-beta central in the unaffected hemisphere (adj *R*^2^ = 0.098); when adding the second variable (model 2), high-beta central in the affected hemisphere, we observed an improvement of the model (adj *R*^2^ = 0.148). Finally, when we added MT (model 3), we noticed an important increase in the adj *R*^2^ (0.366) suggesting that MT is the best predictor for motor function. Details of the models are reported in Table 3.

**Table 3:** **Results for multivariate linear regression analyses in which the outcome variable was Fugl-Meyer (FM) and independent variable were EEG power (i.e., high-beta central of the affected and unaffected hemisphere) and motor threshold (MT) of the affected side**.

FM	β coefficient	*p* Value
**Model 1—Adj *R*^2^ = 0.098**
High beta unaffected hemisphere	60.34	0.012
**Model 2—Adj *R*^2^ = 0.148**
High beta unaffected hemisphere	172.43	0.005
High beta affected hemisphere	−101.52	0.046
**Model 3—Adj *R*^2^ = 0.366**
High beta unaffected hemisphere	122.40	0.020
High beta affected hemisphere	−97.71	0.028
MT affected side	−0.45	<0.001

### Exploratory Analysis—Ratio Affected/Unaffected Hemispheres

**Figure 1:**
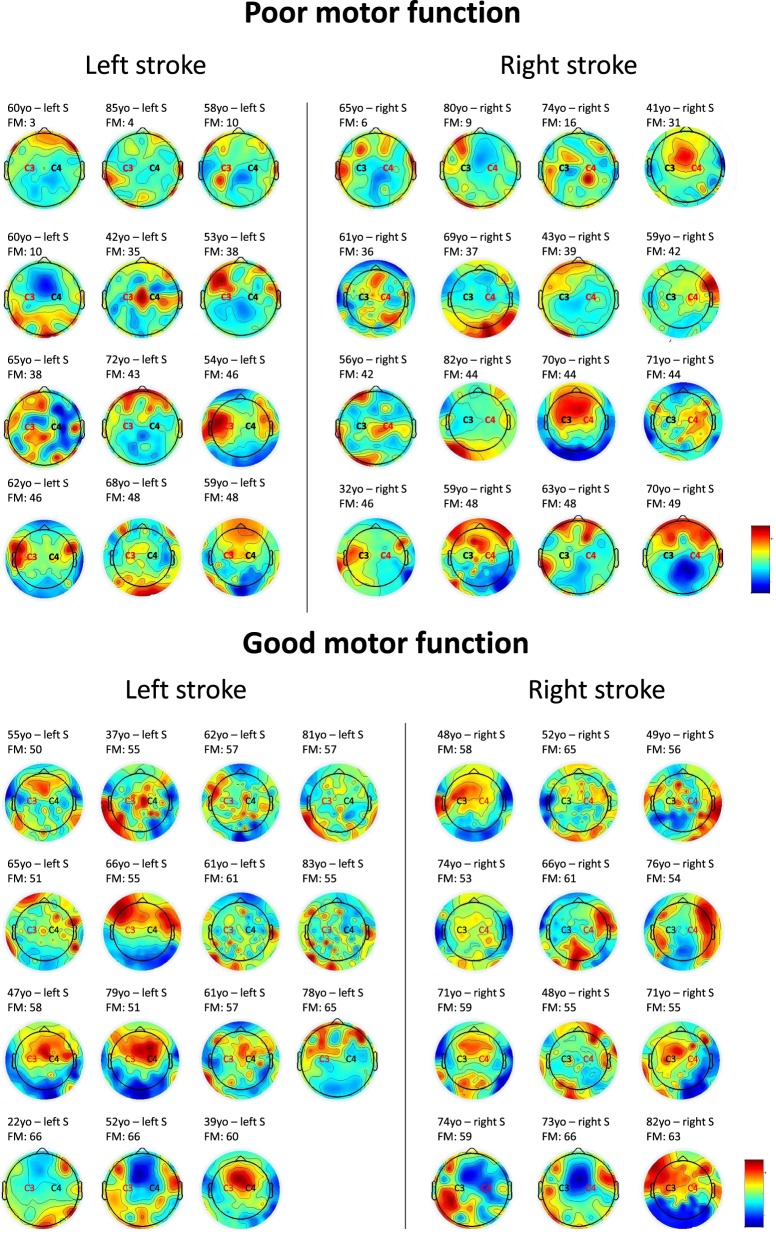
**Topoplots showing the topographic distribution of high-beta bandwidth (25 Hz) for every individual**. Red areas represent higher high-beta activity, while blue areas represent lower high-beta activity. Central region (C3 or C4) in red stands for the affected side. For patients with poor motor function, a higher beta activity of the affected central region as compared to the affected side is observed in 16 out of 28 individuals. For patients with good motor function, a similar activity over central regions bilaterally, or higher activity over the unaffected central area can be identified in 21 out of 27 individuals. FM = Fugl-Meyer.

To investigate the interaction between high beta in the central region of the affected and unaffected hemispheres, we calculated the ratio between affected and unaffected high-beta central for the patients with a good and a poor motor function (above or below the median of FM scores for the 55 patients—median = 49). Twenty-eight patients had a score ≤49 and 27 had a score >49. We observed that patients have better FM scores when the ratio affected/unaffected is equal to 1 or below (i.e., 0.122/0.129 = 0.94), while if the ratio is >1 toward the affected hemisphere (i.e., 0.114/0.095 = 1.194) motor function is poorer—see Figure 1 for individual results. When comparing the proportion of patients with a ratio >1 and patients with a ration ≤1 in the two groups (FM scores ≤ or >49), we identified a significantly higher number of patients with a ratio >1 in the group of low FM scores as compared to the one with higher FM scores (*Z* = −2.0922, *p* = 0.037). Note that we compared the ratio for other bandwidths (i.e., theta and alpha bandwidths) and other brain regions (i.e., frontal and parietal regions of the affected and unaffected hemispheres); this pattern (i.e., ratio >1) was only found for high-beta bandwidth over the central area.

### Confounders

For the above-mentioned models, we further tested the possible confounders. We analyzed the effect of age, time since stroke, gender, side of lesion, type of stroke (ischemic or hemorrhagic), and center use as potential confounders but observed no main effect for any of these variables (age: *p* = 0.172; time since stroke: *p* = 0.506; gender: *p* = 0.274; side of lesion: *p* = 0.399; type of stroke: *p* = 0.375). We then forced the variable “center” (1. Brazil and 2. Italy) into the model and did not find an effect for this variable (*p* = 0.135).

## Discussion

In this cross-sectional study, we identified that EEG biomarkers in the beta frequency over the central regions for both affected and unaffected hemispheres are associated with functional outcome of stroke patients. Based on these results, we proposed some hypotheses that can be further investigated in future trials.

### Relationship between High-Frequency Cortical Activity and Motor Function

Our results confirmed that cortical excitability of the affected cortical side is correlated with the motor impairment in patients with chronic stroke. Likewise, MT was positively associated with the FM score, indicating that higher MT (or decreased corticospinal excitability) is associated with a lower motor function. This result has been previously reported and is in agreement with the view of MT as a biomarker for the amount of the damage in the motor cortex (17). We also found that EEG data increase the capacity of our models to explain the variability of motor function. However, evidence supporting EEG as an independent predictor of adaptive plasticity was scarce before this report.

In this study, we observed a significant correlation between power in the high-frequency bands, including high alpha (10–13 Hz), low beta (13–20 Hz), and high beta (21–30 Hz), and motor impairment. When looking at the EEG variables that explain more variability of the motor function, we found high-beta power in the central electrodes (i.e., C1, C2, C3, C4, C5, C6) of the affected and unaffected hemispheres, but in the opposite direction. In other words, increased high beta over the central region in the affected hemisphere is related to lower FM scores (worse motor function), while, relative increased high beta over the central region in the unaffected hemisphere is related to higher FM scores (better motor function).

This finding suggests that increased high-frequency EEG band power (i.e., beta) in the affected hemisphere is associated with a poor motor function. This could reflect a pathological reorganization with an excess of activity. The excess of activity may reflect the difficulty for a patient to perform a task and also, an attempt of reorganization. Indeed, it has been shown that an increase in beta activity was related to motor tasks requiring higher efforts. For instance, higher beta activity has been observed in the motor cortex in elderly subjects as compared to younger participants or when a task becomes more complex, suggesting that this increase may have a compensatory effect (18–22). As compared to these trials using active tasks, our study looked at brain activity at rest, and not during a specific motor task. However, our results, even if recorded at rest, reflect that an excess of cortical processing is related to a poor motor function. It is important to highlight that we cannot conclude that excessive beta power causes poor motor function as this is a cross-sectional study; but rather that there is an association between the two. In fact, using a simple analogy, increased beta power in poor motor function may be similar to increased insulin levels in hyperglycemia (insulin does not lead to increased glycemic levels but rather tries to compensate).

An important aspect is to try to correlate different neurophysiological tools such as TMS and EEG parameters to build combined markers to understand and predict stroke recovery. In a preliminary analysis, we did not find a correlation between MT and EEG when analyzing both affected and unaffected hemispheres. However, univariate analysis using the affected hemisphere showed a negative correlation between MT of the affected side and high-beta power in the central region of the affected hemisphere. Given that this finding was only observed in the affected hemisphere, this may also be related to the volume of the lesion. Although this analysis did not show a correlation between the affected and unaffected hemispheres, future combined markers may provide important information, as seen in our multivariate model, where the outcome variable was FM. For instance, a previous multimodal study performed in stroke subjects and healthy controls using H2^15^O-positron emission tomography (PET), EEG (during hand movements), and TMS (MT), showed that stroke patients had higher beta coherence in the fronto-central region and higher MT in the affected hemisphere as compared to healthy controls, suggesting a reduction in connectivity of the corticospinal tract in the affected hemisphere (23).

### Interhemispheric Imbalance between the Affected and Unaffected Hemispheres

The balance between beta power of both hemispheres was also a relevant variable to predict motor function. For instance, an index constructed with the beta activity of the affected versus unaffected hemispheres was informative about motor function: patients with a ratio that approached “1” had higher FM scores (i.e., better motor function). This evidence is in line with the hypothesis of interhemispheric imbalance, according to which the activity of the motor cortex that suffers an insult interferes with the activity of the hemisphere in the process of physiological remodeling. Therefore, the interhemispheric beta power ratio could be a marker of maladaptive plasticity. In addition, this could mean that a good motor recovery is related to a normalization of cortical activity in the motor area. Regarding cortical activity in the motor cortex when performing a motor task, an event-related desynchronization of beta-band activity is observed before an active movement. During the preparation and voluntary execution of movements, we can observe a desynchronization of the mu rhythm as well as beta power over the central sensorimotor regions (24, 25), whereas beta-band power increases in scalp EEG data have been related to movement suppression (19). In neurological conditions such as Parkinson’s disease, an excess of beta synchrony is observed and is associated with impaired motor functions (26, 27). This observation is in line with our interpretation that an excess of beta activity in the affected hemisphere is related to a poor motor function and could be interpreted as a maladaptive increase in cortical activity.

As aforementioned, we observed two opposite outcomes regarding beta activity in the affected and unaffected central regions. A relative increase in this frequency band within the central region in the unaffected hemisphere is correlated with a good motor function, while an increase in the affected hemisphere is associated with a poor motor function. In addition, when looking at the ratio of these two variables, we observed lower FM scores when beta activity was higher in the affected as compared to the unaffected hemisphere. Therefore, we need to interpret an increase in beta activity in three different ways: (1) as a recovery process, (2) a compensation or maladaptive plasticity, and (3) an indirect marker of the brain lesion. Many neuroimaging studies using task-related functional magnetic resonance imaging (fMRI) or PET-scan to evaluate brain activity and its correlation with motor recovery after stroke were done. For instance, patients with a good outcome demonstrated a brain activity closer to the physiological region (e.g., left supplementary motor area for a movement of the right hand), while for patients with a poor outcome, the contra-lateral region was activated when performing the requested task [e.g., right supplementary motor area for a movement of the right hand—Nelles et al. (28, 29)]. A normalization of the physiological brain activity after a stroke seems to be a good predictor of motor recovery. As compared to fMRI and PET-scan, EEG represents a more affordable option with the advantage of being transportable and used at patients’ bedside. That is why, future studies using resting-state EEG, as in the present study, and active paradigms, as potential predictive markers of stroke recovery, should be performed in order to confirm the possible value of EEG in clinical practice to predict good or poor motor recovery.

### Clinical Translation

Another question we need to answer is “how this finding could help or improve rehabilitation strategies?” We can potentially use this brain oscillatory pattern as a reorganization index; meaning that a beta-power index (affected/unaffected) higher than “1” means poor cortical reorganization and interventions may be tested on basis of this index. Another way in which our findings could improve rehabilitation strategies is through neurofeedback. Likewise, patients can learn through operant conditioning the patterns of beta oscillations that produce better motor outcomes (30). The former intervention, as well as non-invasive brain stimulation, may prime the motor cortex before a therapy is applied. In addition, our findings identified an excess of activity within the affected motor cortex, with an imbalance between the two hemispheres. This maladaptive reorganization has shown to play a key role in poor functional outcomes (31, 32). By stimulating the physiological pathways, rehabilitation strategies or non-invasive brain stimulation could help reducing the maladaptive excess of activity toward a more balanced activity between the two hemispheres, and therefore, improve patients’ recovery. In addition, it will be important to evaluate whether these findings could be translated to EEG systems used in clinical practice. For instance, the clinical system BIS (Bispectral Index™) used in anesthesiology to monitor depth of anesthesia, was first developed using high-density EEG and was further adapted for clinical use. Similar procedures should be done to translate quantitative EEG findings using high-density EEG systems to clinical EEG recordings.

### Limitations

Our results need to be read encompassing some caveats. First, this is not a longitudinal study evaluating patients’ brain oscillations over time. Therefore, we cannot claim any causal relationship between the excess of beta activity in the affected hemisphere and poor motor recovery. Future trials recording EEGs at different time points during the course of a patient’s recovery should be performed to answer that question. Another limitation is that we used datasets from two different centers. However, when entering the covariate “center” in the model, we showed that it did not influence the results significantly. Noteworthy, by coupling the data from two sites we were able to perform our analyses on a relatively big sample size, which strengthen our results. It should also be noted that we recorded resting-state EEG and not EEG during a specific motor task. Using such active paradigm, for instance, testing event-related desynchronization, could provide alternative findings and be more closely related to motor function. A combination of both active and resting paradigms should be tested in future clinical trials. Regarding clinical data, some medications, especially GABAergic agents (e.g., Baclofen) are known to have an impact on high-frequency cortical activity. Future studies should take into account such medications in order to evaluate how this factor could influence cortical oscillations and its correlation with motor function. However, it is important to note that for some of our findings, we compared hemispheric changes, and we expect a similar effect of these medications on both hemispheres. In addition, the localization and the size of the brain lesion could also have influenced the results. In our study, this information was not systematically assessed, as it was not the main goal of this investigation. Therefore, neuroimaging studies combining EEG with structural MRI (i.e., voxel-based morphometry) could provide further insights into the relationship between motor function, cortical oscillations, and structural damages. As abovementioned, this study was done using high-density EEG recordings and cannot, yet, be translated to clinical practice. Trials using a smaller number of electrodes, as systems used in clinical practice, should confirm the practicability and reproducibility of our results. Finally, these results must be considered preliminary since these exploratory analyses were not corrected for multiple comparisons.

## Conclusion

This study expands our previous findings on the neurophysiological markers of motor function after stroke and proposes some models for using EEG as a potential marker of motor recovery that needs to be validated in longitudinal clinical trials or cohorts. Clinical trials can also test whether interventions that modulate neural activity have an impact on EEG data when comparing before and after the procedure according to the models discussed here. These markers could also identify physiologic phenotypes with a better response to treatment, or in other words, to identify not only surrogate markers but prognostic markers. Its relative inexpensiveness and temporal resolution make EEG an attractive technology to explore brain function in a clinical setting, thus models that integrate several biomarkers will advance further the field of motor recovery after stroke.

## Ethics Statement

This study was carried out in accordance with the recommendations of the “University of Sao Paulo Ethics Committee” and the “University Hospital of Pisa Ethics Committee.” All subjects gave written informed consent in accordance with the Declaration of Helsinki. The protocol was approved by the Ethics Committees of both Universities.

## Author Contributions

MS, FF, CC, and LB designed the study. MS, CF, and FB collected the data. AT, RH-G, and FF analyzed and interpreted the data and wrote the manuscript. All authors critically reviewed the manuscript and approved the final version.

## Conflict of Interest Statement

The authors declare that the research was conducted in the absence of any commercial or financial relationships that could be construed as a potential conflict of interest.
